# Optimize the number of cycles of induction chemotherapy for locoregionally advanced nasopharyngeal carcinoma: a propensity score matching analysis

**DOI:** 10.7150/jca.65315

**Published:** 2022-01-01

**Authors:** YuTing Jiang, KaiHua Chen, Jie Yang, ZhongGuo Liang, Song Qu, Ling Li, XiaoDong Zhu

**Affiliations:** 1 Department of Radiation Oncology, Guangxi Medical University Cancer Hospital, Nanning, China.; 2 Department of Oncology, Affiliated Wuming Hospital of Guangxi Medical University, Nanning, China.

**Keywords:** Nasopharyngeal carcinoma, Induction chemotherapy, Cycle number, Survival

## Abstract

**Background:** There is no conclusive on the optimal number of cycles of induction chemotherapy (IC) with the greatest benefit to patient survival. This study aimed to assess the efficiency and acute toxicities of different cycles of IC for patients with locoregionally advanced nasopharyngeal carcinoma (LA-NPC).

**Methods:** We reviewed data from patients with LA-NPC treated with IC plus concurrent chemoradiation (CCRT). Propensity score matching (PSM) was applied to match paired patients. After PSM, survival outcomes of matched patients were compared between two and three cycles of IC groups. Univariate and multivariate Cox regression analysis were carried out to identify potentially independent predictors. Treatment-related acute toxicities between the two groups were compared by Pearson X^2^ test or Fisher's exact test.

**Results:** In total, 189 pairs were selected. The median follow-up time was 60 months (range 5 to 126 months). There was no difference between two and three cycles of IC in terms of 5-year overall survival (87.0% vs. 89.7%, p = 0.991), distant metastasis-free survival (90.1% vs. 86.8%, p = 0.587), locoregional recurrence-free survival (97.0% vs. 93.8%, p = 0.488), or progression-free survival (79.4% vs. 79.3%, p = 0.896). Multivariate Cox analysis showed that T stage, N stage, and clinical stage were independent prognostic factors. Three cycles of IC were associated with a higher incidence of Grade 1-2 acute toxicity than two cycles during IC period.

**Conclusion:** The efficacy of two cycles of IC achieved similar survival outcomes as three cycles and has a lower incidence of treatment-related acute toxicity.

## Introduction

Nasopharyngeal carcinoma (NPC) is a common malignant tumor with unbalanced geographic distribution, and It is often diagnosed in Southern China, Southeast Asia and North Africa 1. Unlike other head and neck cancers, radiation therapy (RT) is the mainstay of treatment for non-metastatic NPC given its deep-seated anatomical location and high sensitivity to radiation. Among them, more than 70% of patients are classified as locoregionally advanced disease (LA-NPC) and concurrent chemoradiation (CCRT) has been recommended as the standard treatment for them 2-4. The addition of adjuvant chemotherapy (AC) or induction chemotherapy (IC) to CCRT may decrease the risk of distant metastasis and local recurrence in patients, which contribute to a survival benefit. However, AC often has low compliance for three cycles (about 60%) due to a high incidence of adverse events 5. Compared with AC, the addition of IC before radiotherapy is better tolerated and offers advantages for shrinkage of tumor volume and early eradication of micrometastases 5, 6. In the past decade, IC has gained extensive research. Previous studies 6-10 have reported that LA-NPC patients benefited from two, three, and even four cycles of IC. Thus, the latest National Comprehensive Cancer Network (NCCN) Guidelines recommends IC plus CCRT as one of the most appropriate treatments for LA-NPC patients (category IA), which is superior to CCRT (category IIA) and CCRT+AC (category IB).

However, the survival improvement of this aggressive treatment is often accompanied by more toxicities, which may have negative influence on patients' tolerance to subsequent CCRT. Some studies have reported that as compared to CCRT alone, the addition of IC to CCRT increase the incidence of Grade 3 or 4 side events 6, 11. As IC plus CCRT has been recommended as an effective treatment modality, more detailed and effective IC decisions (number of cycles, regimen, dose, etc.) with less adverse events are needed. Although most randomized controlled trials use three cycles of IC, about 3%-25% of patients do not successfully complete three cycles due to toxicity and treatment costs 6, 12-14. There is no conclusive on the number of IC cycles with the greatest benefit to patient survival. Previous studies have shown that additional cycles of IC after two failed to increase survival rate for patients with LA-NPC compared with two cycles of IC, but increase a higher incidence of treatment-related toxicity 15-17. It is important to identify optimal number of IC cycles in daily clinical practice. The current study aimed to explore the optimal number of cycles of IC in LA-NPC patients receiving IC and CCRT.

## Materials and methods

### Patients

From January 2010 to June 2018, a total of 655 patients in our hospital were enrolled in this retrospective study. The inclusion criteria of this study included: (1) newly histologically proved stage III-IVa NPC (restage based on 8th edition of the AJCC/UICC staging system); (2) treated with two or three cycles of IC plus CCRT; (3) no history of anti-tumor treatment before our study; (4) available clinical data, examination information and follow-up data; (5) no serious diseases or secondary malignancy when diagnosed as NPC. Of 655 patients, 189 pairs (57.7%) were matched for the present study. This retrospective study was approved by the Medical Ethics Committee of our hospital. The need to obtain informed consent was not required as this was a retrospective study.

### Quantification of plasma EBV DNA level

The detailed measurement of EBV DNA level was reported by our previous study 18, The cutoff value for pretreatment EBV DNA level (pre-EBV DNA) was set at 5000 copies/mL, which was calculated by receiver operator characteristic (ROC) curve analysis.

### Treatment

All patients received treatment based on the institutional guidelines' recommendation. The regimens of IC consisted of TPF, TP, PF, and GP regime, which were administered every three weeks for 2 to 4 cycles before CCRT. Concurrent chemotherapy was cisplatin regimen (80-100 mg/m2) for 1 to 3 cycles. Treatment-related acute toxicities were classified by the Common Toxicity Criteria for Adverse Events version 4.0 (CTCAE 4.0).

All patients underwent radical IMRT. Detailed chemotherapy and radiotherapy treatment information is in accordance with the principles of our previous studies and shown in **Supplementary Materials** 18, 19.

### Follow-up

Patients received outpatient examination or telephone to conduct follow-up after treatment. All patients were regularly screened with physical examination, nasopharyngoscopy, and imaging every three months in first two years after RT, six months in the next three years, and annually thereafter until death. The overall survival (OS) was the main endpoint. The secondary endpoints included distant metastasis-free survival (DMFS), locoregional relapse-free survival (LRRFS), and progression-free survival (PFS). OS was defined as the date from histological diagnosis to death or last follow-up. DMFS was defined as the date from histological diagnosis to first distant metastasis or last follow-up visit. LRRFS was defined as the date from histological diagnosis to first locoregional relapse or last follow-up visit. PFS was defined as the date from histological diagnosis to first treatment failure, death, or last follow-up visit.

### Statistical analysis

The statistical analyses were executed using SPSS (version 25.0) and R software (version 3.6.3). To minimize the influence of selection bias by potential confounding factors, 1 : 1 propensity score matching (PSM) was applied to compare baseline clinicopathological characteristics between IC = 2 and IC = 3 groups, with the nearest neighbor-matching method and a caliper of 0.05 (by the “Matchlt” package in R). Categorical variables were classified according to clinical knowledge, and numerical variables (age and cumulative cisplatin dose) were transformed to categorical variables based on the findings reported in previous studies 19-21. Categorical variables were presented as whole numbers and proportion. The Pearson X^2^ test or Fisher's exact test was carried out to evaluate the differences in proportions of patients' baseline characteristics and acute toxicity between the two groups. Kaplan-Meier analysis and the log-rank test were carried out to calculate survival rates and compare the differences between the two groups (by the “survival” package in R). Univariate and multivariate Cox regression analysis were used to identify potentially independent prognostic factors by a backward stepwise algorithm. Variables selected by the univariate Cox analyses (p < 0.05) were included into the multivariable Cox analyses. Hazard ratios (HRs) and 95% confidence intervals (CIs) were recorded to indicate the prognostic value of risk factors.

Two-sided P-values < 0.05 was considered to be statistically significant.

## Results

### Patients characteristics

A total of 655 LA-NPC patients who met the eligibility criteria were enrolled into this study. Of those, there were 466 cases in IC = 3 group and 189 in IC = 2 group. Although not all patients in this study had pre-EBV DNA data, we still included it in propensity score matching (PSM) analysis because it might be a confounding factor. Overall, 189 pairs were selected according to the matching analysis. **Table 1** summarized the patients' clinicopathological characteristics. The distribution of age, sex, smoking history, pathology, pre-EBV DNA, T stage, N stage, clinical stage, and cumulative cisplatin dose between the two groups were not statistically significant different (all P > 0.05). Compared with the IC = 2 group, the IC = 3 group had more patients accepted TPF or GP regime and less patients treated with PF regime (78.8% vs. 66.7%, 10.6% vs. 1.6%, and 5.3% vs. 27.5%, respectively, P < 0.001).

### Survival outcome

**Figure 1:**
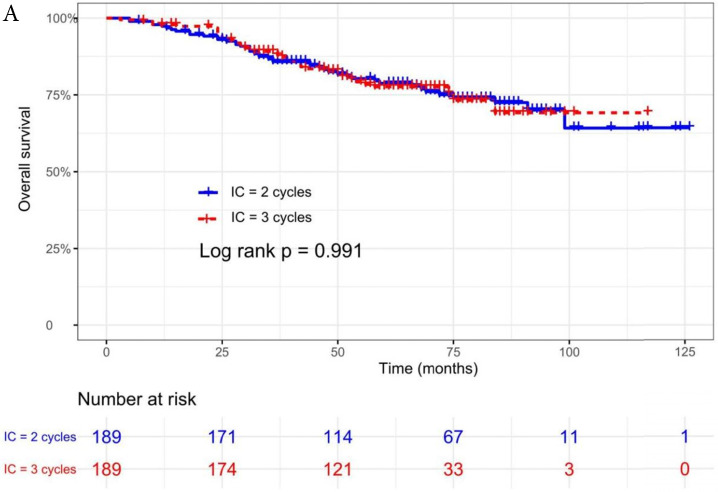

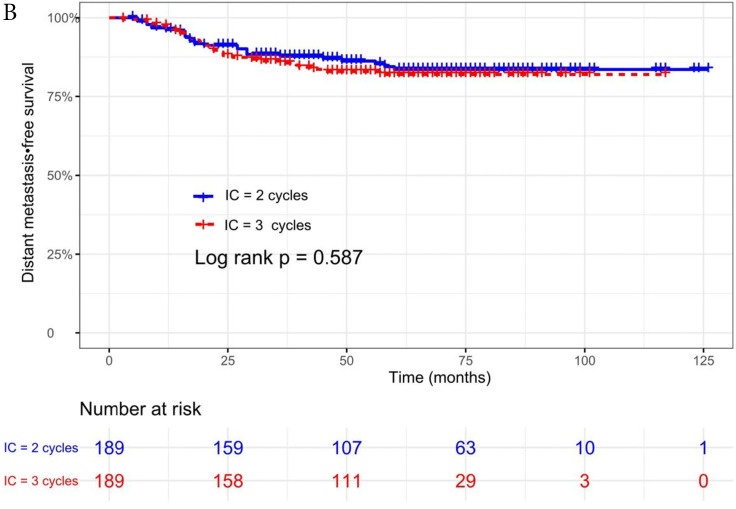

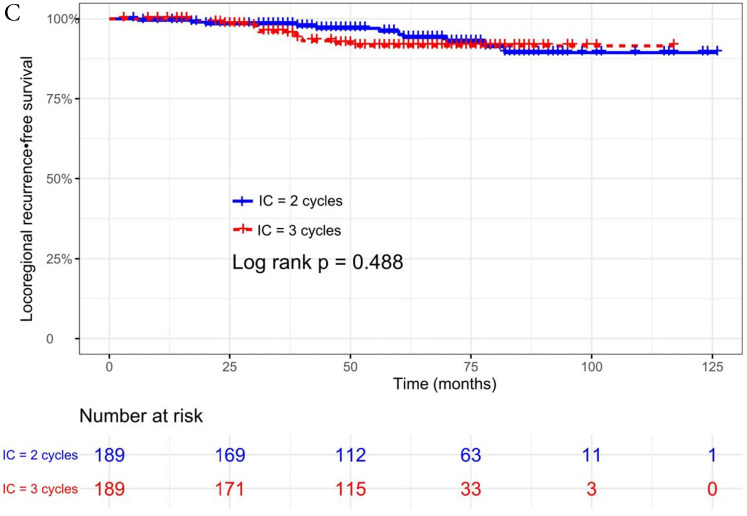

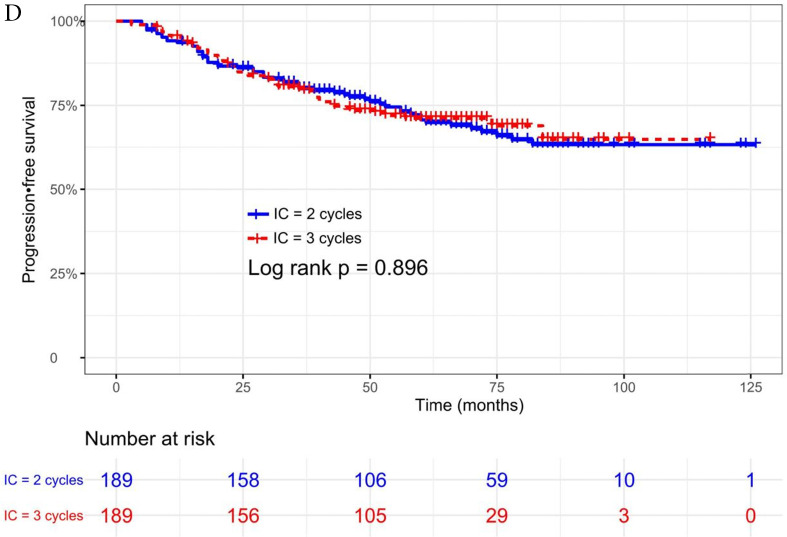
Kaplan-Meier OS (A), DMFS (B), LRRFS (C) and PFS (D) curves for 189 pairs of patients stratified as IC = 2 cycles and IC = 3 cycles groups. Abbreviations: OS, overall survival; DMFS, distant metastasis-free survival; LRRFS, locoregional recurrence-free survival; PFS, progression-free survival.

The median follow-up time was 60 months (range 5 to 126 months). The 5-year OS, DMFS, LRFS, and PFS in IC = 2 group and IC = 3 group were 83.9%, 87.7%, 97.0%, 79.4% and 83.5%, 85.6%, 93.8%, 79.3% respectively. Up to the last follow-up, 44 patients (23.3%) in IC = 2 group and 40 patients (21.1%) in IC = 3 group died; 27 patients (14.3%) in IC = 2 group and 31 patients (16.4%) in IC = 3 group experienced distant metastasis; 11 patients (5.8%) in IC = 2 group and 13 patients (6.9%) in IC = 3 group developed locoregional recurrence; 57 patients (30.2%) in IC = 2 group and 52 patients (27.5%) in IC = 3 group developed treatment failure. No statistically significant difference in OS, DMFS, LRFS, and PFS was detected between two IC cycles groups (all P > 0.05; **Fig. 1** and** Table 2**).

### Identification of prognostic factors

We did univariate analysis about the candidate variables that might be predictors based on basic theoretical knowledge, clinical significance, and predictors confirmed by previous studies, the variables included gender (male vs. female), age (≥50 years vs. <50 years), smoking history (yes vs. no), pathology (WHO III vs. WHO I/II), T stage (T3, T4 vs. T1-2), N stage (N2, N3 vs. N0-1), clinical stage (Stage IVa vs. Stage III), pre-EBV DNA (≥ 5000 copies/ml vs. < 5000 copies/ml), IC regimen (TP, PF, GP vs. TPF), and cumulative cisplatin dose (≥ 200 mg/m^2^ vs. < 200 mg/m^2^). Concrete Results of univariate analysis are presented in **Table 3**. According to univariate analysis, the variables associated to lower OS were gender (male); advanced age, T stage, N stage, clinical stage; and higher pre-EBV DNA (≥ 5000copies/ml) (all P < 0.05). The following variables were not correlated with treatment failure: smoking history; pathology; IC regime, cumulative cisplatin dose, and number of IC cycles. Compared to 2 cycles of IC, additional more cycles (IC = 3) could not significantly improve OS (HR, 0.997; 95% CI, 0.648-1.536; P = 0.991), DMFS (HR, 1.153; 95% CI, 0.688-1.932; P = 0.588), LRRFS (HR, 1.329; 95% CI, 0.592-2.983; P = 0.490) and PFS (HR, 0.975; 95% CI, 0.669-1.422; P = 0.897; **Table 3** and **Fig. 1**).

The variables included in multivariate analysis were prognostic factors identified by univariate analysis. Through PSM, there was still significant difference of IC regime in the different IC cycles group (**Table 1**). Thus, we also included IC regime into the multivariate analysis to test whether it was an independent prognostic factor. The outcomes of the multivariate analysis revealed that IC regime was not associated with significantly improved survival rates. The visual details of this step for the OS, DMFS, LRRFS and PFS were shown in **Table 4**. By means of Spearman correlation analysis, we found that T stage and N stage were correlated with clinical stage, with the correlation coefficients of 0.486 (p < 0.001) and 0.421 (p < 0.001), respectively. Thus, clinical staging didn't be included in multivariate analysis in **Table 4**, and we conducted another multivariate analysis combined clinical staging and other prognostic factors besides T, N stage in **Supplementary Table 1**. According to multivariate analyses, T stage, N stage, and clinical stage remained independent prognosticators.

### Subgroup analysis

According to the results of multivariate analyses, patients with advanced T stage, N stage, or clinical stage would associate with higher rate of treatment failure. Then we examined whether patients with different risk stratification would benefit differently for different IC cycles or not, so we conducted subgroup analysis to further examine the treatment efficiency of different IC cycles on different risk subgroups. In addition, pre-EBV DNA was showed to be a predictor by univariate analysis in this study. Because not all patients in this study had pre-EBV data, we did not include it in the multivariate analysis. However, previous studies used pre-EBV DNA to conduct risk stratification 22, 23, we also divided patients into two subgroups according to pre-EBV DNA and investigated the role of number of cycles of IC in these two subgroups. The results of subgroup analysis indicated that no significant differences were found between two or three cycles of IC for patients stratified by pre-EBV DNA, T stage, N stage, and clinical stage. Details regarding subgroup analysis of survival outcomes are provided in **Table 5.**

### Acute toxicity

During IC period, complete hematology results were available for all patients and we evaluated the treatment-related acute toxicity between different IC cycles groups. Details of patients with Grade 1-2, and 3-4 acute toxicities are presented in **Table 6**. There was no significant difference in terms of the incidence of Grade 3-4 acute toxicities within the two groups. However, three cycles of IC significantly increased the prevalence of Grade 1-2 leukocytopenia (P < 0.001), neutropenia (p = 0.014), anemia (P = 0.022), hepatoxicity (bilirubin increase, P < 0.001), and gastrointestinal reactions (P < 0.001), as compared to two cycles of IC.

## Discussion

In the present study, we retrospectively analyzed 378 locoregionally advanced NPC with IC and CCRT and revealed the treatment efficiency of the number of cycles of IC through the PSM method. The above findings indicated that the treatment efficacy of two cycles of IC is equivalent to three cycles of IC in LA-NPC, but decrease the incidence of treatment-related Grade 1-2 acute toxicities during the IC period. In addition, the stratified subgroups analysis results demonstrated two cycles of IC offered similar survival benefit over three cycles for patients in both the high-risk group and the low-risk group. These results revealed that two cycles of IC might be sufficient and additional more cycles of IC did not lead to survival benefit.

For non-metastatic LA-NPC, CCRT has been proved as the standard of care according to previous studies 2, 3, 24. With the wide application of intensity-modulated radiotherapy (IMRT), the local control rate of NPC has been significantly improved and the occurrence of distant metastasis has become the main cause for treatment failure 25-27. In recent years, adding IC to CCRT has received considerable attention. A previous randomized phase 2 study reported that the application of IC to CCRT was superior to CCRT alone for 3-year overall survival, and also led to a trend to improve progression-free survival and distant control 8. Later several studies provided evidence that IC plus CCRT could afford a survival benefit for LA-NPC patients 6, 11, 28, and IC has since played an important role of the treatment regimen for LA-NPC. In most randomized trial and clinical practice, physicians may prefer three cycles as initial IC regimen. However, a considerable number of patients could not successfully complete the three cycles IC due to the treatment-related toxicity and cost. It is also unclear whether three is the optimal number of IC cycles for maximizing patient survival or not. Considering the toxicity and economic cost of IC, accurate judgment of optimal number of cycles of IC merits further study.

Theoretically, LA-NPC patients with advanced stage are likely to have a higher risk of occurrence of disease progression. Some of them would develop subclinical micrometastasis at initial of diagnosis, which indicates they may benefit from intensive treatment. Although it is not recommended as a high level of evidence for application in clinical practice, clinicians may choose more cycles of IC for these “high-risk” subgroups. In the current study, we identified which variables would affect survival outcomes and assessed the prognostic value of these variables by univariate and multivariate Cox analyses. We found that T stage, N stage, and clinical stage were independent prognostic factors of OS, DMFS, LRRFS, or PFS, and the number of IC cycles was not a predictor of any survival outcome. Thus, according to the prognostic factors chosen by multivariate analysis with different tumor burden and risk of treatment failure, we conducted subgroups analysis to further investigate the efficacy of cycles of IC. The results of this step showed that non-significant differences were observed in all endpoints between different IC cycles groups. These results indicated that additional more cycles after two could not add benefit for patients with higher pre-EBV DNA or advanced stage.

Our study showed that three cycles IC could not improve OS, DMFS, LRRFS, or PFS in LA-NPC patients, which were consistent with previous study by Wei et al 16. The subgroup analysis further demonstrated no survival difference between the patient subgroups, regardless of pre-EBV DNA or tumor stage. We speculated there were two reasons for this situation. One reasonable explanation for the finding is that two cycles of IC may be sufficient for LA-NPC patients to eradicate micrometastases and decrease tumor volume. Another potential reason why the difference was not significant is the prolonged waiting time for radiotherapy (WRT) by additional IC cycles. WRT was defined as the interval between cancer diagnosis and the implementation of radical radiotherapy. Generally, oncologists think that early radical treatment is reasonable and cancer patients should receive radical treatment as soon as possible after a definite diagnosis. However, timely treatment is hampered in many patients due to lack of policy support, limited medical resources, or inefficient healthcare process 29. Some medical strategy-related factors are also important factors in clinical practice, especially IC, which is one of the main treatment-related factors prolonging WRT. The association between prolonged WRT and poor prognosis has been found in many cancers, such as breast cancer, rectal cancer, and bladder cancer 30-33. Also, a number of previous retrospective studies suggested prolonged WRT was correlated with worse survival outcome for NPC patients. Chen et al reported that an interval time of > 4 weeks between diagnosis and radical radiotherapy was an independent negative predictor for PFS 34. Another later study with a larger population and longer follow-up indicated that increased WRT correlated with poor clinical outcome of NPC patients with advanced stage 29. The above studies reminded us that IC could prolong WRT and may decrease survival rate even though it has been proven to ameliorate clinical outcomes for NPC patients. NPC patients receiving three cycles of IC usually experience longer WRT than that of patients treated with only two cycles, which may “counteract” the benefit of additional cycles of IC. Thus, we suspect that WRT might be a potential confounding variable that subtly influences the results of this study. These hypotheses could explain why this study failed to observe significant differences of treatment efficacy between the different IC cycles groups.

Studies of the optimal number of cycles of IC for LA-NPC patients treated with IC plus CCRT are scarce and their conclusions were controversial. Peng et al evaluated 247 pairs of NPC patients with advanced stage and found no significant difference in survival between patients with 2 cycles and 3 to 4 cycles of IC, while in N2-3 stage subgroup, patients treated with 2 cycles IC had better OS than those of patients with 3 to 4 cycles IC (92.4% vs. 80.8%, P = 0.029) 17. In another retrospective analysis, Wang et al demonstrated that 2 cycles of IC could significantly improve DMFS (HR, 0.499; P=0.038) and PFS (HR, 0.585; P=0.049) compared with 3 to 4 cycles, and the results of stratified analysis indicated that LRRFS, PFS, and OS were comparable between the different IC cycles groups for N0-1 category patients 15. However, both studies defined high cycle group as patient with 3 or 4 cycles of IC and the 3 cycles IC were not explored separately. As we all know, three cycles were usually used in randomized controlled clinical trials and clinical practice. Additionally, the population of the two studies was all staged by the 7th AJCC/UICC staging system, which would lack generalizability in real-world clinical practice with the advent of the 8th staging system. A recent study by He et al compared survival outcome from patients treated with 2, 3 and 4 IC cycles and found similar survival between 2 and 3 cycles IC groups, while 4 cycles IC was associated with worse overall survival and higher incidence of treatment-related toxicities 16. However, the above study also used the 7th staging system and included stage II patients for whom IC is not routinely recommended according to the latest guideline 35. Also, this study set in a non-endemic area of China with higher rate of non-keratinizing diferentiated subtype (approximate 30%) than endemic area (< 5%), and this histological type is an adverse prognostic factor for patient survival 36, 37. Although the results were not entirely consistent, these studies corroborated the fact that additional more cycles of IC might not lead to better survival outcomes. In contrast to these three studies, the scholars in another study reported that the number of IC appeared to be an independent predictor and for N2-3 NPC, survival data of the 4 cycles of IC were better than those of 2 cycles 20. It is noted that the study paid attention to advance N stage patients and lack of patient receiving 3 cycles of IC. These published inconsistent results above mentioned implied that the effect of the number of IC cycles on prognosis is an important problem to be noticed and requires additional research. According to most findings of above studies, we speculated that the treatment efficacy of three cycles of IC may not be superior to two cycles and our results supported this hypothesis. Compared to other studies, our study paid attention to stage III-IVa NPC in endemic area of China and performed based on the 8th AJCC/UICC staging system. Our finding may provide better guidance for treatment decisions in areas with high incidence of NPC.

During the period of IC, the most commonly observed acute adverse events included hematologic toxicities and gastrointestinal reactions. Our study showed that additional more cycles increase the incidence of treatment-related Grade 1-2 toxicity. Although Grade 1-2 acute toxicity was acceptable and easily managed, patients may be affected by it for subsequent CCRT. In a randomized controlled trial, only 30% of patients with three cycles IC completed three cycles of concurrent cisplatin successfully 6. In clinical practice, the most common reasons that patients do not continue concurrent cisplatin are patients' refusal and treatment-related toxicities. If patient experienced too much adverse events during IC period, patients' fear of acute adverse events may decrease their tolerance to subsequent CCRT.

In this study, we used PSM to evaluate the treatment efficiency of two and three IC cycles for patients with LA-NPC, which increase the reliability of the results. However, several limitations should be stated. First, this is a retrospective study, and inherent selective bias was unavoidable. The IC regimen was not balanced among the IC = 2 and IC = 3 group because there were no standard IC regimens at the time and the determination of the treatment decision would take individual patient's situation into account. This factor might be a confounding factor when evaluated survival rates. Of note, the multivariate analyses including this factor could effectively reduce this bias. In this study, IC regime failed to be an independent prognostic factor for survival outcomes. Additionally, all regimens included in this study (TPF, TP, PF, GP) were platinum-based and recommended according to the guideline, which have been widely used in many hospitals 16, 18, 21. Second, the data from a single institution do not provide robust evidence. Therefore, these results must be validated by other institutions.

## Conclusion

In summary, our retrospective study indicated that two cycles of IC appear to provide similar survival benefit over additional more cycles for patients with LA-NPC and may be associated with lower incidence of treatment-related adverse events. This finding will help patients avoiding overtreatment and financial burden. Additionally, patients may have better compliance to CCRT if appropriate cycles of IC were administered. Based on the above evidence, we believe that two cycles of IC may be more reasonable for patients with LA-NPC than three cycles. This conclusion needs to be confirmed by multi-center prospective study with large cohort.

## Supplementary Material

Supplementary methods and table.

## Figures and Tables

**Table 1:** Baseline clinical characteristics.

Characteristics	n (%)		p-value
	IC = 2 (n = 189)	IC = 3 (n = 189)	
Age (year)			0.742
< 50	60 (31.7)	63 (33.3)	
≥ 50	129 (68.3)	126 (66.7)	
Sex			0.737
Female	59 (31.2)	56 (29.6)	
Male	130 (68.8)	133 (70.4)	
Smoking			
No	137 (72.5)	140 (74.10	0.727
Yes	52 (27.5)	49 (25.9)	
Pathology			
WHO I/II	20 (11.8)	20 (11.8)	1.000
WHO III	169 (88.2)	169 (88.2)	
T stage			0.261
1	1 (0.5)	5 (2.6)	
2	53 (28.1)	43 (22.8)	
3	83 (43.9)	90 (47.6)	
4	52 (27.5)	51 (27.0)	
N stage			
0	1 (0.5)	1 (0.5)	0.629
1	44 (23.3)	40 (21.2)	
2	103 (54.5)	96 (50.8)	
3	41 (21.7)	52 (27.5)	
Clinical stage			0.216
III	106 (56.1)	94 (49.7)	
IVa	83 (43.9)	95 (50.3)	
pre-EBV DNA (copies/ml)			0.096
NA	27 (14.3)	5 (2.6)	
< 5000	92 (48.7)	88 (46.6)	
≥ 5000	70 (37.0)	96 (50.8)	
IC regimen			
TPF	126 (66.7)	149 (78.8)	<0.001
TP	8 (4.2)	10 (5.3)	
PF	52 (27.5)	10 (5.3)	
GP	3 (1.6)	20 (10.6)	
cumulative cisplatin dose (mg/m^2)^			0.700
≥ 200	149 (78.8)	153 (81.0)	
< 200	40 (21.2)	36 (19.0)	

Note: Data are shown as number of patients (%) or median (IQR). Abbreviations: WHO, World Health Organization; pre-EBV DNA, pretreatment Epstein-Barr virus DNA; TPF, docetaxel plus cisplatin plus 5-fluorouracil; TP, docetaxel plus cisplatin; GP, gemcitabine plus cisplatin; PF, cisplatin plus 5-fluorouracil. NA, these patients had not pre-EBV DNA data.

**Table 2:** Efficacy of Study Treatment.

Survival outcomes	IC = 2 (n = 189)	IC = 3 (n = 189)	p-value
Overall survival			0.991
Deaths	44 (23.3)	40 (21.2)	
3 year OS rate (%)	87.0	89.7	
5 year OS rate (%)	83.9	83.5	
Distant metastasis-free survival			0.587
Distant metastasis	27 (14.3)	31 (16.4)	
3 year DMFS rate (%)	90.1	86.8	
5 year DMFS rate (%)	87.7	85.6	
Locoregional recurrence-free survival			0.488
Recurrence	11 (5.8)	13 (6.9)	
3 year LRRFS rate (%)	98.3	95.9	
5 year LRRFS rate (%)	97.0	93.8	
Progression-free survival			0.896
Failures	57 (30.2)	52 (27.5)	
3 year PFS rate (%)	85.0	83.9	
5 year PFS rate (%)	79.4	79.3	

Abbreviations: OS, overall survival; DMFS, distant metastasis-free survival; LRRFS, locoregional recurrence-free survival; PFS, progression-free survival.

**Table 3:** Prognostic factors on survival outcomes of 378 LA-NPC patients by use of univariate analysis.

Characteristics	OS		DMFS		LRRFS		PFS	
	HR (95% CI)	P	HR (95% CI)	P	HR (95% CI)	P	HR (95% CI)	p
Age (year)		0.025		0.567		0.877		0.359
< 50	Reference		Reference		Reference		Reference	
≥ 50	1.656 (1.066-2.572)		0.845 (0.475-1.504)		1.072 (0.443-2.595)		1.205 (0.809-1.793)	
Sex		0.005		0.421		0.711		0.142
Female	Reference		Reference		Reference		Reference	
Male	2.209 (1.263-3.863)		1.273 (0.707-2.291)		1.181 (0.489-2.854)		1.386 (0.897-2.142)	
Smoking		0.199		0.898		0.605		0.498
No	Reference		Reference		Reference		Reference	
Yes	1.355 (0.852-2.155)		0.962 (0.535-1.732)		0.605 (0.207-1.772)		1.155 (0.761-1.753)	
Pathology		0.878		0.636		0.642		0.821
WHO I/II	Reference		Reference		Reference		Reference	
WHO III	0.947 (0.474-1.893)		0.826 (0.375-1.821)		0.750 (0.224-2.517)		1.078 (0.563-2.067)	
T stage		0.049		0.261		0.762		0.048
T1-2	Reference		Reference		Reference		Reference	
T3	1.070 (0.608-1.070)	0.814	1.664 (0.831-3.331)	0.151	1.008 (0.366-2.773)	0.988	1.107 (0.678-1.809)	0.684
T4	1.814 (1.026-3.208)	0.041	1.810 (0.855-3.832)	0.121	1.383 (0.480-3.987)	0.548	1.731 (1.050-2.854)	0.031
N stage		<0.001		<0.001		0.076		<0.001
N0-1	Reference		Reference		Reference		Reference	
N2	1.555 (0.770-3.142)	0.218	2.406 (0.924-6.266)	0.072	0.389 (0.150-1.009)	0.052	1.393 (0.778-2.495)	0.265
N3	5.103 (2.540-10.254)	<0.001	6.418 (2.467-16.99)	<0.001	1.099 (0.406-2.979)	0.852	4.936 (2.451-7.884)	<0.001
Overall stage		<0.001		<0.001		0.094		<0.001
III	Reference		Reference		Reference		Reference	
IVa	3.724 (2.296-6.043)		2.679 (1.547-4.640)		2.007 (0.888-4.534)		3.082 (2.052-4.628)	
pre-EBV DNA (copies/ml)		0.043		0.035		0.902		0.026
< 5000	Reference		Reference		Reference		Reference	
≥ 5000	1.655 (1.037-2.642)		1.817 (1.043-3.167)		1.054 (0.456-2.434)		1.575 (1.052-2.356)	
IC regimen		0.637		0.549		0.979		0.421
TPF	Reference		Reference		Reference		Reference	
TP	1.113 (0.405-3.058)	0.836	1.108 (0.345-3.555)	0.863	1.131 (0.150-8.505)	0.905	1.366 (0.596-3.128)	0.461
PF	0.958 (0.543-1.688)	0.881	0.540 (0.231-1.261)	0.154	1.230 (0.453-3.339)	0.685	0.916 (0.550-1.525)	0.736
GP	0.276 (0.038-1.998)	0.202	NA	0.927	1.210 (0.158-9.267)	0.854	0.357 (0.087-1.454)	0.150
IC cycle		0.991		0.588		0.490		0.897
IC = 2	Reference		Reference		Reference		Reference	
IC = 3	0.997 (0.648-1.536)		1.153 (0.688-1.932)		1.329 (0.592-2.983)		0.975 (0.669-1.422)	
cumulative cisplatin dose (mg/m^2)^		0.352		0.435		0.315		0.867
≥ 200	Reference		Reference		Reference		Reference	
< 200	1.282 (0.760-2.162)		0.753 (0.370-1.534)		1.609 (0.637-4.065)		1.042 (0.647-1.678)	

Abbreviations: WHO, World Health Organization; pre-EBV DNA, pretreatment Epstein-Barr virus DNA; TPF, docetaxel plus cisplatin plus 5-fluorouracil; TP, docetaxel plus cisplatin; GP, gemcitabine plus cisplatin; PF, cisplatin plus 5-fluorouracil; OS, overall survival; DMFS, distant metastasis-free survival; LRRFS, locoregional recurrence-free survival; PFS, progression-free survival; CI, confidence interval; HR, hazard ratio.

**Table 4:** Prognostic factors on survival outcomes of 378 LA-NPC patients by use of multivariate analysis.

Endpoints	Variable	HR (95% CI)	p-value
OS	Age (≥ 50 vs. < 50)	1.161 (0.739-1.824)	0.516
	Sex (Male vs. Female)	1.782 (0.999-3.177)	0.051
	T stage (3 vs. 1-2)	1.268 (0.714-2.252)	0.417
	T stage (4 vs. 1-2)	2.228 (1.230-4.035)	0.008
	N stage (2 vs. 0-1)	1.840 (0.899-3.766)	0.095
	N stage (3 vs. 0-1)	6.074 (2.952-12.497)	<0.001
	IC regimen (TPF vs. others)	1.334 (0.797-2.230)	0.273
DMFS	Age (≥ 50 vs. < 50)	0.735 (0.419-1.289)	0.283
	Sex (Male vs. Female)	1.099 (0.598-2.021)	0.761
	T stage (3 vs. 1-2)	2.109 (1.045-4.259)	0.037
	T stage (4 vs. 1-2)	2.758 (1.262-6.027)	0.011
	N stage (2 vs. 0-1)	3.106 (1.108-8.178)	0.022
	N stage (3 vs. 0-1)	9.191 (3.452-24.477)	<0.001
	IC regimen (TPF vs. others)	2.021 (0.989-4.132)	0.054
LRRFS	Age (≥ 50 vs. < 50)	0.919 (0.381-2.218)	0.851
	Sex (Male vs. Female)	1.109 (0.445-2.767)	0.824
	T stage (3 vs. 1-2)	0.780 (0.262-2.319)	0.655
	T stage (4 vs. 1-2)	1.051 (0.322-3.425)	0.935
	N stage (2 vs. 0-1)	0.377 (0.135-1.058)	0.064
	N stage (3 vs. 0-1)	1.091 (0.371-3.212)	0.874
	IC regimen (TPF vs. others)	0.851 (0.350-2.066)	0.721
PFS	Age (≥ 50 vs. < 50)	1.974 (0.654-1.452)	0.897
	Sex (Male vs. Female)	1.111 (0.707-1.744)	0.648
	T stage (3 vs. 1-2)	1.348 (0.817-2.225)	0.242
	T stage (4 vs. 1-2)	2.356 (1.389-3.999)	0.001
	N stage (2 vs. 0-1)	1.696 (0.935-3.075)	0.082
	N stage (3 vs. 0-1)	5.584 (3.046-10.237)	<0.001
	IC regimen (TPF vs. others)	1.220 (0.787-1.891)	0.374

Abbreviations: OS, overall survival; DMFS, distant metastasis-free survival; LRRFS, locoregional recurrence-free survival; PFS, progression-free survival; CI, confidence interval; HR, hazard ratio.

**Table 5:** Subgroup analysis.

Characteristic	OS (%)			DMFS (%)			LRRFS (%)			PFS (%)		
	IC=2	IC=3	P	IC=2	IC=3	P	IC=2	IC=3	P	IC=2	IC=3	P
T stage												
T1-2	81.3	84.2	0.743	92.2	84.8	0.296	97.4	93.5	0.753	77.1	78.6	0.855
T3	81.5	79.3	0.773	82.0	80.6	0.625	95.5	90.2	0.199	73.6	70.9	0.571
T4	72.2	68.5	0.978	77.7	81.4	0.610	92.1	91.7	0.568	60.1	64.9	0.395
N stage												
N0-1	95.1	84.9	0.617	93.1	95.0	0.702	97.4	83.3	0.194	88.3	77.4	0.596
N2	80.8	85.7	0.488	83.4	87.7	0.541	95.8	97.5	0.263	73.9	83.0	0.182
N3	52.8	56.8	0.810	69.0	61.1	0.268	80.8	87.7	0.468	36.4	44.5	0.735
Clinical stage												
III	88.7	90.2	0.651	88.6	90.8	0.691	97.0	93.8	0.666	81.8	85.9	0.423
IVa	65.5	65.4	0.875	76.3	72.8	0.522	91.3	88.9	0.732	53.9	58.2	0.984
pre-EBV DNA (copies/ml)												
< 5000	77.7	85.0	0.401	84.7	90.6	0.447	93.1	92.8	0.817	69.7	81.6	0.153
≥ 5000	75.5	72.4	0.937	81.2	74.9	0.312	98.2	90.1	0.214	67.8	63.3	0.647

Abbreviations: pre-EBV DNA, pretreatment Epstein-Barr virus DNA; OS, overall survival; DMFS, distant metastasis-free survival; LRRFS, locoregional recurrence-free survival; PFS, progression-free survival.

**Table 6:** Acute toxicity in patients during IC.

Variable	IC = 2 (n = 189)	IC = 3 (n = 189)	p-value
Haematological			
Leukocytopenia			
Grade 1/2	65	102	<0.001
Grade 3/4	23	18	0.408
Neutropenia			
Grade 1/2	47	69	0.014
Grade 3/4	33	28	0.485
Thrombocytopenia			
Grade 1/2	8	10	0.629
Grade 3/4	3	4	1.000
Anemia			
Grade 1/2	30	48	0.022
Grade 3/4	1	2	1.000
Hepatoxicity			
ALT increase			
Grade 1/2	53	70	0.131
Grade 3/4	5	5	1.000
AST increase			
Grade 1/2	42	57	0.079
Grade 3/4	3	1	0.623
Bilirubin increase			
Grade 1/2	20	57	<0.001
Grade 3/4	0	0	1.000
Gastrointestinal reactions			
Grade 1/2	75	109	<0.001
Grade 3/4	2	4	0.685

Note: All data are presented as number of patients (%). Abbreviations: ALT, alanine aminotransferase; AST, aspartate transaminase.
