# Supplementary material for: Optimize the number of cycles of induction chemotherapy for locoregionally advanced nasopharyngeal carcinoma: a propensity score matching analysis
Source: J Cancer. 2022 Jan 1;13(2):426–35. doi: 10.7150/jca.65315 (PMC8771525; doi:10.7150/jca.65315)
Supplement: Supplementary file 1 — Supplementary methods and table. [file jcav13p0426s1.pdf]

supplementary file:

Supplementary Table 1 Prognostic factors on survival outcomes of 378 LA-NPC patients by use of multivariate analysis besides T and N stage

Endpoints	Variable	HR (95% CI)	p-value
OS	Age (≥ 50 vs. < 50)	1.053 (0.712-1.737)	0.797
	Sex (Male vs. Female)	1.116 (0.717-1.737)	0.628
	Clinical stage (IVa vs. III)	3.010 (1.990-4.552)	<0.001
	IC regimen (TPF vs. others)	1.121 (0.720-1.743)	0.613
DMFS	Age (≥ 50 vs. < 50)	0.785 (0.451-1.366)	0.392
	Sex (Male vs. Female)	1.084 (0.597-1.968)	0.792
	Clinical stage (IVa vs. III)	2.679 (1.534-4.679)	<0.001
	IC regimen (TPF vs. others)	1.899 (0.932-3.872)	0.077
LRRFS	Age (≥ 50 vs. < 50)	0.963 (0.405-2.288)	0.931
	Sex (Male vs. Female)	1.039 (0.422-2.555)	0.934
	Clinical stage (IVa vs. III)	2.015 (0.878-4.622)	0.098
	IC regimen (TPF vs. others)	0.801 (0.331-1.938)	0.622
PFS	Age (≥ 50 vs. < 50)	1.121 (0.720-1.743)	0.797
	Sex (Male vs. Female)	1.116 (0.717-1.737)	0.628
	Clinical stage (IVa vs. III)	3.010 (1.990-4.552)	<0.001
	IC regimen (TPF vs. others)	1.121 (0.720-1.743)	0.613

Abbreviations: OS, overall survival; DMFS, distant metastasis-free survival; LRRFS, locoregional recurrence-free survival; PFS, progression-free survival; CI, confidence interval; HR, hazard ratio.

Supplementary materials:

Details of treatment

The regimens of IC included TPF regimen (docetaxel with cisplatin with 5-fluorouracil, 60, 60, and 3,000 mg/m2, respectively), or TP regimen (docetaxel with cisplatin, 75 and 75 mg/m2, respectively), or GP regimen (gemcitabine with cisplatin, 1000 and 80 mg/m2, respectively), or PF regimen (cisplatin with 5-fluorouracil, 80 and 4,000 mg/m2, respectively).

The primary gross tumor volume (GTVnx) and cervical lymph node tumor volume (GTVnd) included the entire macroscopic tumor defined with the aid of computed tomography (CT), magnetic resonance imaging (MRI) scans, and physical examinations. Two clinical target volumes (CTVs) were delineated according to the tumor invasion pattern. The high-risk clinical target volume (CTV1) included the GTVnx add a margin of 0.5 to 1 cm (forward, both sides, up and down) and a margin of 0.3 to 0.5 cm (back) to encompass the high-risk sites of microscopic extension and the whole nasopharynx. The low-risk clinical target volume (CTV2) was defined as the CTV1 add a margin of 0.5 to 1 cm (forward, both sides, up and down) and a

margin of 0.3 to 0.5 cm (back) to encompass the low-risk sites of microscopic extension, the GTVnd, and elective neck area from level IB to V. The prescribed doses to the PTVs of GTVnx, GTVnd, CTV1, and CTV2 were 70.0~74.0 Gy/31~33 f, 60.0~73.6 Gy/30~33 f, 60.0~64.0 Gy/30~32 f, and 54.0~60.0 Gy/30~32 f, respectively.
